# Clonal hematopoiesis of indeterminate potential: a multisystem hub bridging hematopoietic dysfunction with non-hematopoietic diseases

**DOI:** 10.1186/s40779-025-00654-8

**Published:** 2025-10-14

**Authors:** Jing-Lei Zhang, Shuo-Lan Tong, Qi-Qi Zhuang, Sheng-Jie Jin, Jia-Qiu Li, Jie Sun

**Affiliations:** 1 Department of Oncology, Affiliated Hospital of Shandong Second Medical University, Shandong Second Medical University, Weifang, 261042 Shandong China; 2 Zhejiang Key Laboratory for Precision Diagnosis and Treatment of Hematological Malignancies, Hangzhou, 310006 China; 3 Department of Hematology, the First Affiliated Hospital, Zhejiang University School of Medicine, Hangzhou, 310006 China; 4 Department of Biochemistry and Molecular Biology, Monash University, Clayton, Melbourne, VIC 3168 Australia; 5 Clinical Research Center, Affiliated Hospital of Shandong Second Medical University, Shandong Second Medical University, Weifang, 261042 Shandong China; 6 Zhejiang Provincial Clinical Research Center for Hematological Disorders, Hangzhou, 310006 China

**Keywords:** Clonal hematopoiesis of indeterminate potential (CHIP), Chronic inflammation, Atherosclerosis, Solid tumors, Infection

## Abstract

Clonal hematopoiesis of indeterminate potential (CHIP), driven by leukemia-related somatic mutations in hematopoietic stem cells, previously recognized as a major risk factor for hematological malignancies, has now emerged as a potent risk factor for chronic inflammation and diverse non-hematologic diseases. CHIP-associated DNA methyltransferase 3 alpha *(DNMT3A)*, tet methylcytosine dioxygenase 2 *(TET2)*, and additional sex combs like 1 *(ASXL1)* mutations alter epigenetic programs, skew myelopoiesis, and increase proinflammatory cytokines, resulting in chronic inflammation and immune imbalance. This review integrates mechanistic insights with clinical evidence to delineate CHIP’s roles in solid tumors, cardiovascular disorders, and metabolic dysregulation, with an extended discussion of renal dysfunction and neurodegenerative conditions. Furthermore, we also discuss CHIP’s diagnostic and therapeutic impacts across multiple disease contexts, advocating for mutation-specific diagnostic paradigms to guide therapeutic interventions.

## Background

Somatic mutations in pre-leukaemia driver genes within hematopoietic stem/progenitor cells (HSPCs) accumulate over time, with some mutations conferring a selective growth advantage to HSPCs, which is referred to as clonal hematopoiesis (CH) [1, 2]. When these mutations are detected with a variant allele frequency (VAF) greater than 2% in blood or bone marrow, but without hematological abnormalities, the condition is classified as clonal hematopoiesis of indeterminate potential (CHIP) [3]. These somatic mutations promote clonal dominance by enhancing HSPC proliferation, thereby reshaping the hematopoietic hierarchy [4, 5]. Although CHIP was initially recognized as a precursor state for hematologic malignancies, accumulating evidence implicated it as a systemic risk factor for diverse non-hematologic diseases via immune dysregulation and chronic inflammation [4, 6, 7].

Although CHIP sub-clones are traditionally defined by a minimum VAF threshold of 2% [3, 5], advances in next-generation sequencing (NGS) have revealed that clones below this threshold may retain clinical significance [3, 8]. Despite their smaller clonal size, these low-VAF clones are associated with elevated risks of cardiovascular and metabolic disorders [9–11], potentially via similar proinflammatory mechanisms as larger clones. The clinical relevance of low-VAF clones challenges the sufficiency of existing VAF-based criteria and suggests a need for their reassessment.

The occurrence of CHIP is primarily associated with aging, environmental exposures, and genetic susceptibility. Age is the most significant risk factor, with a detection rate of 10% in individuals over 65 years old and only 1% in those under 50 years old, suggesting that hematopoietic stem cells (HSCs) acquire somatic mutations [e.g., DNA methyltransferase 3 alpha (*DNMT3A*), tet methylcytosine dioxygenase 2 (*TET2*), additional sex combs like 1 (*ASXL1*)] due to cumulative DNA damage, which serves as the key driving mechanism [4, 6]. Environmental factors such as smoking and obesity further promote mutations through oxidative stress and an inflammatory microenvironment: smoking may primarily promote the expansion of *ASXL1*-mutant clones indirectly by altering the inflammatory microenvironment [12–15], while high-fat diets activate bone marrow inflammatory pathways, providing a proliferative advantage to mutated clones [16]. Moreover, DNA damage repair stress induced by cancer treatments (e.g., chemotherapy) can amplify mutated clones, such as tumor protein p53 (*TP53)* and protein phosphatase magnesium-dependent 1D, thereby increasing the risk of secondary hematologic cancers [17–19]. These factors collectively lead to CHIP, which essentially is the adaptive competition of mutated HSCs in the bone marrow microenvironment, accompanied by sustained activation of inflammatory pathways such as NF-κB [20, 21].

**Fig. 1:**
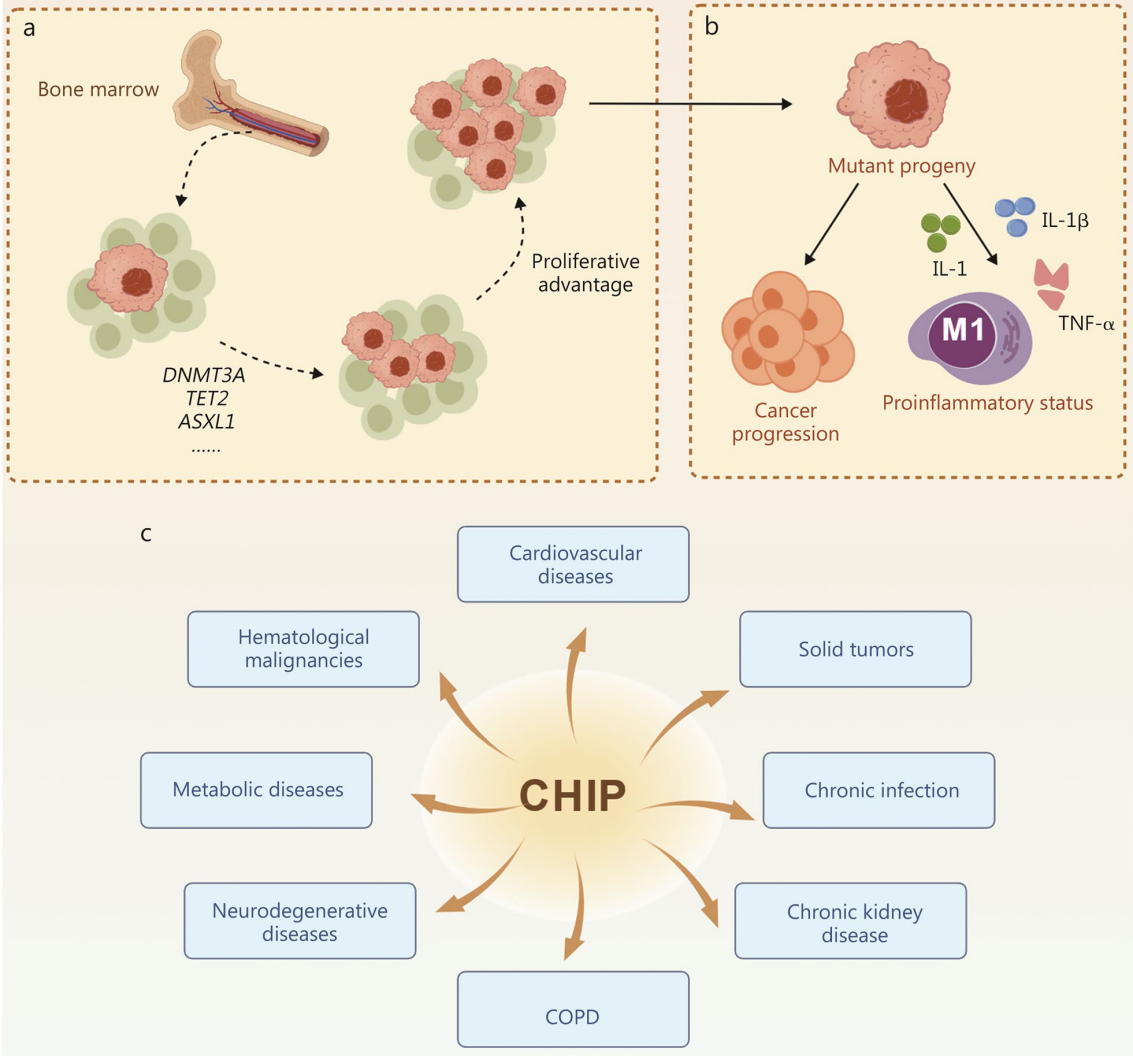
Mechanistic modules and systemic impacts of CHIP. **a** CHIP-related mutations and clonal expansion: somatic mutations in epigenetic regulators (e.g., *DNMT3A*, *TET2*, *ASXL1*) occur in HSPCs, conferring clonal expansion. **b** Inflammatory and immune alterations: mutant progeny promote proinflammatory responses, including IL-1β, IL-6, and TNF-α secretion and M1 macrophage polarization, driving systemic immune dysregulation. **c** Systemic disease manifestations: CHIP-associated inflammation contributes to diverse pathologies across multiple organ systems, including cardiovascular diseases, chronic obstructive pulmonary disease (COPD), metabolic disorders (e.g., type 2 diabetes, gout), chronic kidney/liver disease, solid tumors, hematological malignancies, neurodegenerative diseases, and chronic infection. CHIP clonal hematopoiesis of indeterminate potential, DNMT3A DNA methyltransferase 3 alpha, TET2 tet methylcytosine dioxygenase 2, ASXL1 additional sex combs like 1, HSPCs hematopoietic stem/progenitor cells, IL-1β interleukin-1β, IL-6 interleukin-6, TNF-α tumor necrosis factor-α. Created with BioGDP.com

Persistent inflammation driven by CHIP-associated mutations serves as the primary mechanism promoting CHIP-associated disease progression, as these mutations disrupt DNA repair, epigenetic regulation, and transcriptional homeostasis, thereby skewing macrophage polarization toward proinflammatory states [22–24]. These alterations led to elevated secretion of interleukin (IL)-1β, IL-6, and tumor necrosis factor-α (TNF-α), promoting vascular inflammation, insulin resistance, and impaired tissue repair, thereby aggravating the progression of cardiovascular diseases (CVDs) [20, 25], therapy-related secondary cancers [25], and metabolic disorders such as type 2 diabetes (T2DM) [26, 27] and potentially to neurodegenerative and chronic conditions including Alzheimer’s disease (AD) [28], chronic kidney disease (CKD) [29], cirrhosis [30], and sepsis mortality [31] (Fig. 1).

## CHIP-associated mutations

Genetically, while more than 70 genes have been implicated in CHIP [13, 32], over 75% of cases involve mutations in just 4 genes: *DNMT3A*, *TET2*, *ASXL1*, and Janus kinase 2 (*JAK2*) [33]. Although these genes have distinct molecular roles, they collectively contribute to chronic inflammation seen in CHIP. Specifically, these mutations can lead to an imbalance in cytokine production and immune dysregulation, which further exacerbates the systemic inflammatory environment and promotes the development of CHIP-related diseases. Therefore, understanding the genetic basis of CHIP, particularly the roles of these 4 genes, is essential for elucidating how chronic inflammation is sustained and how it contributes to the development of various disease outcomes.

*DNMT3A* encodes a de novo DNA methyltransferase that is essential for transcriptional regulation. The *DNMT3A* R882 hotspot mutation, present in 70–80% of *DNMT3A*-mutated acute myeloid leukemia (AML) cases, impairs enzymatic activity, causing global hypomethylation and site-specific epigenetic alterations (e.g., *Homeobox B2*, *Homeobox B4*). It also leads to transcriptional silencing of differentiation-associated genes (e.g., *Spi-1* proto-oncogene) partly via aberrant Polycomb repressive complexes 1 recruitment, independent of DNA methylation [34, 35]. Moreover, *DNMT3A* R882 mutants (e.g., R882H/C) directly reduce Meis1 locus methylation, promoting its aberrant expression in leukemia models [34]. These epigenetic alterations may trigger innate immune pathways, including cyclic GMP-AMP synthase–stimulator of interferon genes-mediated interferon signaling [35, 36], thereby enhancing HSCs’ self-renewal [37].

*TET2* promotes DNA demethylation by converting 5-methylcytosine to 5-hydroxymethylcytosine, an activity that antagonizes *DNMT3A*’s methyltransferase function [38, 39]. *TET2* mutations induce enhancer hypermethylation (up to 25% of active enhancer elements), contributing to the deregulation of oncogenic transcriptional networks [40]. In the innate immune system, *TET2* deficiency may augment NLR family pyrin domain-containing 3 (NLRP3) inflammasome activation in macrophages, leading to elevated secretion of proinflammatory cytokines, such as IL-1β and IL-6, thereby exacerbating systemic inflammatory responses [41]. Notably, *TET2* plays a dual regulatory role in immune homeostasis: in tumor microenvironments, *TET2* inactivation induces M1-like anti-tumor polarization of macrophages, while *TET2* deficiency simultaneously disrupts the stability of regulatory T cells (Tregs), leading to impaired immune tolerance [42, 43]. Specifically, the combined loss of *TET2*/*TET3* causes hypermethylation at conserved non-coding sequences within the Forkhead box protein P3 locus, potentially driving the conversion of Tregs into proinflammatory T follicular helper cells/T helper 17 cells-like cells [44]. This process attenuates immunosuppressive capacity while promoting chronic inflammation and autoimmunity.

*ASXL1* regulates chromatin remodeling via Polycomb complexes, forming a functional complex with BRCA1-associated protein 1 (BAP1) to catalyze histone H2A lysine 119 monoubiquitination, deubiquitination, and preserve PRC2-mediated transcriptional repression. Notably, when *ASXL1* function is disrupted by mutations, its ability to maintain this chromatin regulatory balance is impaired, and loss-of-function mutations reduce histone H3 lysine 27 trimethylation deposition, derepressing oncogenic programs while activating protein kinase B/mammalian target of rapamycin (Akt/mTOR) signaling to drive the clonal expansion of HSCs [45]. Resultant mitochondrial dysfunction and reactive oxygen species (ROS) accumulation trigger Toll-like receptor 4/myeloid differentiation primary response 88/interleukin-1 receptor-associated kinase signaling cascades, establishing a chronic inflammatory microenvironment [46–48]. Notably, *ASXL1* mutations frequently co-occur with *TET2*, RUNX family transcription factor 1 *(RUNX1)*, SET binding protein 1 *(SETBP1)*, and NRAS proto-oncogene *(NRAS)* mutations, synergistically contributing to disease pathogenesis [49–51].

*JAK2* encodes a non-receptor tyrosine kinase that transduces cytokine signaling through the Janus kinase-signal transducer and activator of transcription (JAK-STAT) pathway. The somatic *JAK2* V617F mutation, the most frequent *JAK2* alteration, resulted in JAK-STAT activation and sustained production of proinflammatory cytokines such as IL-6 and TNF-α, establishing a self-perpetuating inflammatory environment [52, 53]. In macrophages, *JAK2* V617F drives increased erythrophagocytosis, leading to hemoglobin catabolism, iron deposition, and oxidative stress, which contribute to endothelial injury and the development of atherosclerotic and thrombotic lesions [54, 55]. Clinically, a higher *JAK2* V617F allele burden correlated with increased venous thrombosis risk, especially in primary myelofibrosis and high-risk polycythemia vera patients [56]. Pharmacologic inhibition with JAK inhibitors such as ruxolitinib mitigated disease progression in murine models of myeloproliferative neoplasms. It also reduces thrombotic complications and neutrophil extracellular trap formation [57, 58].

## CHIP and solid tumors

### Association of CHIP with solid tumors

#### Pan-cancer epidemiological and mechanistic

CHIP is commonly observed in patients with solid tumors, with prevalence rising with age, and epidemiological studies consistently demonstrate that CHIP occurs more frequently in those with non-hematopoietic cancers than in cancer-free populations, suggesting a potential biological link between CH and solid malignancies [18, 59, 60].

Coombs et al. [18] analyzed paired tumor-blood sequencing data from 8810 cancer patients in a landmark study, demonstrating that mutations in *TET2*, *DNMT3A*, *ASXL1*, and splicing factor 3B subunit 1 *(SF3B1)* exhibited significant enrichment in tumor specimens of patients over 60 years after adjusting for confounding variables, a pattern consistently observed across multiple cancer types. However, such associations may reflect age-related parallel processes rather than a direct causal role of CHIP. To explore potential causality, recent Mendelian randomization analyses have attempted to assess whether CHIP-associated germline genetic predisposition increases the risk of solid tumors. These studies support a potential causal role of CHIP in selected cancers [e.g., non-melanoma skin cancer (NMSC), lung cancer], while evidence for a causal relationship in others, such as prostate cancer, is inconsistent and remains under debate [61–63].

Furthermore, CHIP-associated mutations have been detected in tumor-infiltrating immune cells, raising the possibility that CHIP clones may shape the tumor immune microenvironment. For example, large-scale genomic profiling of 113,079 solid tumors revealed that the most frequently mutated CHIP genes, *DNMT3A*, *TET2*, and *ASXL1*, are often found in infiltrating hematopoietic cells within the tumor microenvironment rather than in tumor cells themselves [60]. Moreover, analyses from the Tracking Non-Small Cell Lung Cancer Evolution through Therapy (TRACERx) cohort have identified tumor-infiltrating clonal hematopoiesis (TI-CH) as a phenomenon frequently observed in CHIP-positive malignancies (Table 1) [14, 18, 25–27, 29–31, 43, 61, 62, 64–83], where it represents a localized manifestation of CHIP-associated clones within the tumor microenvironment [64]. Single-cell analyses reveal that TI-CH mutations (*TET2, DNMT3A, ASXL1*) are enriched in CD206⁺ myeloid subsets (e.g., macrophages), and *TET2*-mutant myeloid cells secrete elevated IL-1β and IL-6, which are implicated in promoting tumor progression [64]. Based on the above discussion, CHIP exhibits a close yet complex relationship with the initiation and progression of solid tumors. This association is not only reflected in genetic co-occurrence but also in the biological process whereby CHIP clones actively reshape the tumor immune microenvironment and thereby promote tumor progression. Thus, CHIP is increasingly recognized not merely as a concomitant phenomenon but as an active participant in cancer biology.

**Table 1:** Summary of the epidemiological association between CHIP and multisystem diseases

Disease		CHIP prevalence (%)	Risk vs. Gen Pop^*^	Associated mutated genes	Risk ratio (*HR* or *OR*)	Key findings	References
Solid tumor							
Pan-cancer		25.1 (CH)^a^; 24 (CHIP)^a^	↑	*DNMT3A*, *TET2*, *PPM1D*, *ASXL1*, *ATM*,* TP53*	–	CHIP is common in solid tumor patients and is linked to age, radiotherapy, and smoking	[18, 64]
Breast cancer		21 (CH)^a^; 15 (CHIP)^b^; 15.1 CHIP)^c^	↑	*DNMT3A*, *TET2*,* TP53*	*HR* = 1.30; Mortality: *HR* = 1.38	CHIP independently increases breast cancer incidence and mortality; Chemotherapy may induce low-VAF *TP53* clones	[18, 65, 66]
Colorectal cancer		–	–	*TET2*,* DNMT3A*	Advanced disease: *HR* = 3.78; Mortality: *HR* = 1.45	CHIP carriers show increased late‐stage diagnosis and mortality, yet the UK Biobank finds no overall risk link to high VAF	[65, 67, 68]
Lung cancer		27 (CH)^a^; 34 (CHIP)^d,e^	↑	*TET2*, *DNMT3A*,* ASXL1*	*HR* = 1.40; VAF ≥ 10%: *HR* = 1.61^d^; Relapse or death: *HR* = 1.42^d,e^	CHIP independently increases lung cancer risk; Mutations in *DNMT3A* and *ASXL1* are the main driving factors	[18, 62, 64, 69]
Prostate cancer		–	↑	*DNMT3A*, *TET2*,* ASXL1*	*HR* = 1.18; *OR* = 1.20	CHIP carriers (particularly *DNMT3A* mutations) increase prostate cancer risk via PI3K/Akt and Wnt pathway dysregulation	[61, 62, 70]
NMSC		–	↑	*DNMT3A*	*HR* = 1.14; *OR* = 1.26	*DNMT3A* mutations promote keratinocyte proliferation; MR supports the causal relationship between CHIP and NMSC	[62]
Melanoma		–	–	*TET2*	*OR* = 1.39	*TET2* deficiency may suppress tumor progression via M1 macrophage expansion	[43, 62]
Cardiovascular diseases							
ASCVD		5.1	↑	*TET2*,* SF3B1/SRSF2/U2AF1*	*HR* = 1.24; VAF ≥ 10%: *HR* = 1.38	CHIP (especially large clones and *TET2*/spliceosome mutations) independently predicts adverse ASCVD outcomes	[25]
CMD		–	↑	*TET2*,* DNMT3A*	*OR* = 3.86	CHIP prevalence is higher in CMD; CHIP mediates 32% of MACE risk	[71]
AF		–	↑	*TET2*, *ASXL1*, *JAK2*,* PPM1D*	*HR* = 1.11; VAF ≥ 10%: *HR* = 1.14	CHIP predicts AF risk independent of coronary artery disease or heart failure; *TET2* mutations confer the highest risk (*HR* = 1.22)	[72]
PAD		–	↑	*TET2*,* DNMT3A*	*HR* = 1.58; VAF ≥ 10%: *HR* = 1.97	CHIP mutations are enriched in atherosclerotic lesions (88% concordant with peripheral blood)	[73, 74]
Kidney disease							
AKI		23	↑	*TET2*, *JAK2*, *ASXL1*, *PPMID*, *TP53*,* SRSF2*	*HR* = 1.34; *OR* = 1.26	Non-*DNMT3A* mutations amplify AKI risk and severity via macrophage-driven inflammation	[29]
CKD		23	↑	*TET2*, *DNMT3A*,* ASXL1*	*HR* = 2.5	CHIP carriers are more likely to progress to end-stage renal disease and exhibit severe anemia	[75]
T2DM		6	↑	*TET2*,* ASXL1*	*HR* = 1.23	Macrophage-driven proinflammatory response leading to insulin resistance	[27, 76]
Osteoporosis		5.7	↑	*DNMT3A*	*HR* = 1.44	Upregulated IL-20 secretion enhances osteoclast activity and reduces bone density	[77]
Gout		5.9	↑	*DNMT3A*,* TET2*	*OR* = 1.69; VAF ≥ 10%: *OR* = 1.25	NLRP3 inflammasome activation with IL-1β secretion promotes urate crystal deposition	[26]
Chronic liver disease		4–9^f^	↑	*TET2*	*OR* = 2.01	Inflammatory cytokine secretion causes organ fibrosis	[30]
COPD		5.7	↑	*DNMT3A*, *TET2*,* ASXL1*	*OR* = 1.6^ g^; *OR* = 2.2^ h^	Chronic inflammatory response exacerbates airway and alveolar structural damage	[14, 78]
HIV		7	↑	*DNMT3A*, *TET2*,* ASXL1*	*OR* = 1.77	Impaired antiviral immunity and enhanced chronic inflammation	[31, 79]
COVID-19		37	↑	*DNMT3A*,* TET2*	*HR* = 1.254	Impaired antiviral immunity and enhanced chronic inflammation	[80, 81]
Neurodegenerative diseases		3.1	↑	*DNMT3A*, *ASXL1*,* SRSF2*	*HR* = 1.10	Excessive production of inflammatory cytokines promotes cerebral atherosclerosis and induces neuronal inflammation and damage	[82]
Periodontitis		3.9	↑	*DNMT3A*	–	Increased osteoclastogenesis and elevated expression of proinflammatory cytokines	[83]

Only diseases with clearly reported epidemiological measures (e.g., *HR*, *OR*, incidence) from cohort studies were included. *Disease risk in CHIP carriers vs. the general population. ^a^MSK-IMPACT cohort. ^b^In early breast cancer. ^c^In metastatic triple-negative breast cancer. ^d^NSCLC patient cohort. ^e^In the TRACERx study. ^f^FHS, *n* = 4230; ARIC, *n* = 7414; UK Biobank, *n* = 201,409; MGBB, *n* = 239,316. ^g^For GOLD 2–4 COPD. ^h^For GOLD 3–4 COPD. *Gen Pop* general population, *DNMT3A* DNA methyltransferase 3 alpha, *TET2* tet methylcytosine dioxygenase 2, *ASXL1* additional sex combs like 1, *PPM1D* protein phosphatase, Mg^2^⁺/Mn^2^⁺ dependent 1D, *ASXL1* additional sex combs like 1, *ATM* ataxia telangiectasia mutated, *TP53* tumor protein p53, *NMSC* non-melanoma skin cancer, *MR* mendelian randomization, *ASCVD* atherosclerotic cardiovascular disease, *SF3B1* splicing factor 3B subunit 1, *SRSF2* serine and arginine rich splicing factor 2, *U2AF1* U2 small nuclear RNA auxiliary factor 1, *CMD* coronary microvascul ar dysfunction, *AF* atrial fibrillation, *JAK2* Janus kinase 2, *PAD* peripheral artery disease, *AKI* acute kidney injury, *CKD* chronic kidney disease, *T2DM* type 2 diabetes, *NLRP3* NLR family pyrin domain containing 3*, COPD* chronic obstructive pulmonary disease, *HIV* human immunodeficiency virus, *COVID-19* coronavirus disease 2019, *CHIP* clonal hematopoiesis of indeterminate potential, *FHS* Framingham Heart Study, *NSCLC* non-small cell lung cancer, *TRACERx* Tracking Non-Small Cell Lung Cancer Evolution through Therapy, *VAF* variant allele frequency, *MACE* major adverse cardiovascular events, *IL* interleukin, *HR* hazard ratio, *OR* odds ratio, *ARIC* atherosclerosis risk in communities, *GOLD* global initiative on obstructive lung disease, *MGBB* Mass general brigham biobank *PI3K/Akt* phosphoinositide 3-kinase/protein kinase B

#### Heterogeneity across tumor types and therapies

CHIP exhibits marked heterogeneity across solid tumors, with prevalence varying by malignancy types (Table 1). A retrospective study identifies thyroid cancer as having the highest CH incidence and germ cell tumors as the lowest [18]. However, population-specific differences have been observed: a pan-cancer analysis of 10,000 Chinese patients reported that thyroid malignancies exhibited the lowest CHIP-related variant frequency [84]. This divergence may be partially explained by therapeutic exposure, given that radiation exposure, particularly radioactive iodine therapy, exhibits dose-dependent relationships with CHIP risk [18, 85]. Such variability implies that CHIP’s influence on clinical outcomes may depend heavily on tissue context.

In addition to differences in tumor types, cancer treatments can also shape the clonal dynamics of CHIP and contribute to its heterogeneity. For example, chemotherapy improves survival in early-stage breast cancer but simultaneously increases the risk of therapy-related myeloid neoplasms (t-MN) [86, 87]. Pre-existing CHIP clones are a major risk factor for t-MN; 30–70% of t-MN cases harbored these clones at their initial cancer diagnosis [88]. Notably, the link between chemotherapy and elevated t-MN risk does not rely on the induction of new CHIP mutations. Instead, chemotherapy may exert a selective pressure that promotes the expansion of pre-existing low-frequency CHIP clones. This aligns with recent data indicating that neither chemotherapy nor endocrine therapy significantly induces new CHIP mutations in early-stage breast cancer patients [66]. However, low-frequency clones (VAF 0.5–2%), especially *TP53*, may selectively expand post-chemotherapy [66]. Intriguingly, even *TP53* mutations with low VAF may hold clinical significance, as these minor clones can expand under chemotherapy-induced selective pressure [89, 90].

#### Clinical impact in specific solid tumors

Given the prevalence of CHIP and its complex interactions with therapies, elucidating its specific impact on the development and prognosis of various solid tumors is crucial. For instance, the association of CHIP with increased breast cancer risk and mortality, as well as with advanced-stage diagnoses and reduced survival in colorectal cancer patients, is likely mediated by its ability to cultivate a pro-tumorigenic microenvironment (Table 1) [65]. The impact on lung cancer appears more complex; while initial large cohort studies found no significant association, subsequent meta-analyses demonstrate that CHIP elevates lung cancer risk, an effect that is particularly pronounced in carriers of large clones (VAF ≥ 10%) (Table 1) [65, 69]. Furthermore, the observation that CHIP with putative driver mutations (CH-PD) is more frequent in lung cancer patients may reflect the shared etiological pressure of smoking, which acts as a mutagen on both HSCs and lung tissues [69]. CHIP and CH-PD are also associated with prior radiotherapy but not chemotherapy [18]. Prospective data confirm CHIP’s independent association with lung cancer, persisting even after excluding chronic obstructive pulmonary disease (COPD) and adjusting for smoking and other risk factors [61, 62]. These discrepancies underscore context-dependent CHIP effects mediated by tumor biology and clonal dynamics, which are critical considerations for precision oncology strategies.

The case of skin cancer further supports a causal role of CHIP, particularly *DNMT3A* mutations, in the pathogenesis of cutaneous malignancies. CHIP demonstrates pathogenic links to cutaneous malignancies. Cohort studies associate CHIP carriers with elevated susceptibility to NMSCs (Table 1), including basal and squamous cell carcinoma [62]. This association appears to be largely driven by *DNMT3A* mutations that promote keratinocyte overgrowth through epigenetic dysregulation [62]. Beyond these epidemiological associations, mechanistic investigations uncovered multifaceted and sometimes contrasting pathobiological crosstalk. For instance, myeloid-specific *TET2* knockout may enhance M1 macrophage polarization and antitumor activity in melanoma models, whereas combined *TET2/DNMT3A* deficiency may augment myeloid-mediated tumor suppression through enhanced interferon-γ signaling [43].

The association between CHIP and prostate cancer remains controversial. Studies suggest that large CHIP clones (VAF ≥ 10%) may confer elevated prostate cancer risk, with *DNMT3A* mutations showing an independent correlation to disease susceptibility (Table 1) [61, 62]. However, a study in European-ancestry males reported no significant link between CHIP and overall or aggressive prostate cancer [63]. These discrepancies may stem from tumor biology, treatment exposure, or genetic background differences.

Future longitudinal and mechanistic studies, especially integrating CHIP clonal dynamics, immune phenotypes, and treatment exposure, are needed to clarify whether CHIP is a bystander, biomarker, or direct contributor to oncogenesis.

### Mechanisms and clinical challenges of CHIP interference with molecular detection of solid tumors

Notably, somatic CHIP mutations in DNA repair genes (e.g., ataxia telangiectasia mutated (*ATM*), breast cancer type 2 susceptibility protein (*BRCA2*), checkpoint kinase 2 (*CHEK2*) can complicate the detection of tumor-specific mutations and undermine diagnostic accuracy [91, 92], as mutant DNA from apoptotic or necrotic CHIP clones enters circulation and mixes with tumor-derived circulating tumor DNA (ctDNA). The resulting admixture may generate low-frequency mutation signals in plasma (VAF 0.1–2%), which could be misinterpreted as tumor-derived [92, 93]. In a cohort of 69 prostate cancer patients, approximately 10% of those with advanced disease showed CHIP-related mutations in DNA repair genes, which are critical for determining eligibility for poly(ADP-ribose) polymerase inhibitor therapy (e.g., olaparib, rucaparib) [93]. In esophageal cancer, CHIP mutations were present in 23% of patients receiving neoadjuvant therapy; exclusion of CHIP variants improved postoperative recurrence prediction accuracy from 75 to 90% [94]. Additionally, research involving 38 colorectal cancer patients found that 29% (11/38) carried CHIP-derived mutations, with 3 patients exhibiting persistent postoperative variants that could be mistaken for residual disease [95]. It is estimated that the incidence of CHIP interference in cell-free DNA (cfDNA) testing for solid tumors may range from 5 to 29% [96].

To mitigate the risk of CHIP mutations obscuring oncogenic findings, blood testing using circulating cfDNA is recommended prior to diagnostic or therapeutic decisions; this can be optimized by matched white blood cell sequencing [93], tumor-targeted DNA panels, sequencing of shorter ctDNA fragments, or analysis of mutation patterns (e.g., C > T transitions typical of CHIP) to distinguish true tumor mutations and reduce false positives [97, 98].

CHIP may also interfere with germline genetic testing. In a cohort of over 110,000 clinical germline tests, CHIP-related variants were identified in 0.05% of cases, with *TP53* mutations accounting for 13% (11/84) of these events [99]. According to the 2024 National Comprehensive Cancer Network (NCCN) guidelines for prostate cancer, clinicians relying solely on ctDNA testing, without tissue biopsy confirmation, should be vigilant for potential false positives due to CHIP, especially in genes such as breast cancer susceptibility genes 1 and 2, ataxia telangiectasia mutated, and checkpoint kinase 2 [100]. The guidelines recommend using prostate cancer-validated NGS platforms, in combination with microsatellite instability and mismatch repair immunohistochemistry, to enhance specificity [100]. Overall, these findings highlight that CHIP is a frequent and clinically relevant source of confounding in cfDNA-based assays, underscoring the necessity of integrated analytical strategies to ensure accurate molecular diagnosis and treatment guidance.

## CHIP and CVDs

### Current evidence for clinical relevance of CHIP and CVDs

#### Association with chronic CVDs conditions

In 2014, Jaiswal et al. [6] were the first to establish a link between CH and atherosclerotic CVDs. They observed that somatic mutations promoting clonal expansion became increasingly prevalent with age, especially over age 70, and were independently associated with elevated risks of coronary heart disease and ischemic stroke. Even after adjusting for traditional cardiovascular risk factors, mutation carriers exhibited a 1.9-fold higher risk of coronary heart disease, increased incidence of ischemic stroke, and greater coronary artery calcification [6]. Building on this foundation, Gumuser et al. [25] analyzed whole-exome sequencing data from 13,129 patients with established atherosclerotic CVDs. They confirmed that CHIP is an independent risk factor for recurrent cardiovascular events and all-cause mortality (Table 1). Notably, mutations in *TET2* and splicing factor genes [*SF3B1*, serine and arginine-rich splicing factor 2 *(SRSF2)*, U2 small nuclear RNA auxiliary factor 1 *(U2AF1)*] with high VAF (VAF ≥ 10%) were most strongly associated with adverse outcomes (Table 1) [25].

CHIP has been identified as a systemic contributor to atherosclerosis, extending its impact beyond coronary arteries to peripheral vascular territories [73]. As evidenced by analyses of peripheral artery disease (PAD) cohorts, 2 independent studies showed that CHIP conferred a 1.6-fold increased PAD risk with VAF > 10% amplifying risk to nearly twofold, exhibiting a dose-dependent pattern where higher variant allele fractions correlate with elevated risk (Table 1) [73]. In a separate study of 31 PAD patients, NGS identified mutations in *TET2* and *DNMT3A* as the most frequently affected genes. Notably, a significant proportion of CHIP mutations detected in peripheral blood were also present in atherosclerotic lesions and in perivascular fat and subcutaneous tissue [74], a finding that directly links CHIP to the local pathological processes of atherosclerosis.

Beyond chronic atherosclerosis, CHIP has been implicated in coronary microvascular dysfunction (CMD). In a retrospective study involving 177 patients presenting with chest pain and no prior history of coronary artery disease, CHIP (VAF ≥ 2%) and CH (VAF ≥ 1%) were found to be more prevalent among those with CMD (Table 1). Furthermore, CMD was independently associated with major adverse cardiovascular events (MACE), with CHIP mediating approximately 32% of this risk, 5-times greater than the direct effect of CMD itself [71]. These findings position CHIP as a significant contributor to poor outcomes in patients with microvascular disease.

UK Biobank cohort data demonstrate CHIP’s independent association with cardiac arrhythmias [72], extending beyond its known associations with coronary artery disease and heart failure. Adjusted analyses have revealed that individuals with CHIP exhibit an increased risk of developing atrial fibrillation (AF), with larger clone sizes further amplifying this risk. Moreover, CHIP has been independently associated with a higher incidence of cardiac arrest. Gene-specific evaluation identified *TET2* mutations as the strongest factor (Table 1) [72]. Animal models support these findings, showing that CHIP promotes AF susceptibility in both atherogenic and non-atherogenic contexts, although arrhythmia onset was slower in the latter [11]. Clinically, CHIP carriers are more likely to require antiarrhythmic therapy and experience arrhythmia recurrences, likely due to CHIP-driven inflammation and myocardial fibrosis [72]. These results suggest that CHIP may represent a modifiable target for the prevention and management of arrhythmias.

#### Association with acute cardiovascular events

Accumulating evidence indicates that CHIP contributes to a spectrum of acute cardiovascular events, ranging from acute myocardial infarction (AMI) to heart failure and cardiogenic shock (CS). A European population study found that individuals under age 65 with AMI were more likely to carry CHIP mutations, and those with higher VAF were specifically prone to heart failure with preserved ejection fraction (HFpEF). Conversely, CHIP prevalence did not differ significantly between AMI cases and controls in older individuals [101]. In addition to its association with myocardial infarction, CHIP has been implicated in acute heart failure. In one large cohort, CHIP carriers had a 70% higher risk of developing heart failure and exhibited more frequent and severe cardiovascular symptoms [102]. Similarly, whole-genome sequencing of postmenopausal women showed that CHIP mutations, especially *TET2* mutations, were associated with a 42% increased risk of HFpEF, without a corresponding increase in heart failure with reduced ejection fraction [103].

Moreover, randomized clinical data have further underscored the prognostic implications of CHIP. In a trial of 446 AMI survivors with severe CS, 57% of CHIP carriers died within 30 d of enrollment, compared to 41% of non-carriers. Combined endpoints, including death or severe renal failure, were also more frequent in the CHIP group, indicating poorer short-term outcomes [104]. Moreover, a comparative analysis between patients with CS and those with ambulatory heart failure showed significantly higher CHIP prevalence among those with CS (25.2% vs. 18.3%), which was associated with reduced survival [105].

The connection between CHIP and CVD is biologically plausible, likely rooted in shared mechanisms of chronic inflammation and immune dysregulation [106]. Despite the established association, the precise pathogenic mechanisms remain under investigation, with current research focused on the roles of major CHIP driver genes in CVD progression. Despite these expanding associations, several key questions remain unanswered, including why specific CHIP driver mutations confer differential cardiovascular risks, how clone size and VAF thresholds modulate disease phenotypes, and whether CHIP-related cardiovascular risks are reversible through anti-inflammatory strategies or clonal suppression. These open questions underscore the need for mechanistic insight, which we discuss in the following section.

### The mechanistic links between CHIP mutations and CVD

#### *TET2* and CVDs

The potential association of CHIP mutations, particularly *DNMT3A*, *TET2*, and *ASXL1* mutations, with atherosclerotic CVDs has been identified [107]. A leading hypothesis suggests that mutant hematopoietic cells, especially monocytes and macrophages, acquire proinflammatory phenotypes, increasing cytokine production and other factors that promote vascular injury and remodeling.

Among the CHIP driver genes, *TET2* has been most intensively studied in relation to CVDs. Fuster et al. [41] demonstrated that transplantation of *TET2*-deficient HSPCs into *Ldlr*⁻/⁻ mice resulted in significantly larger atherosclerotic plaques, driven in part by enhanced IL-1β signaling, mediated via increased activation of the NLRP3 inflammasome in *TET2*-deficient macrophages. Specifically, the size of the descending aortic lesions in the *TET2* knockout mice was 2.7 times larger than that in the control group [108, 109]. Importantly, treatment with an NLRP3 inhibitor attenuated plaque formation, highlighting the inflammasome’s causal role in CHIP-mediated atherogenesis [110, 111]. These findings provide evidence that somatic *TET2* mutations in blood cells may contribute causally to the development of atherosclerosis. The animal studies also showed consistent results that mice with *TET2*-deficient mutations developed larger atherosclerosis lesions in the aortic root and aorta [6]. Beyond vascular inflammation, emerging evidence implicates *TET2* deficiency in cardiac dysfunction [11]. Inactivation of *TET2* may increase the propensity for AF, a process in which NLRP3 is required, and also involves abnormal calcium/calmodulin-dependent protein kinase II (CaMKII)-mediated calcium processing. Loss of *TET2* may affect the activity of cardiomyocyte ryanodine receptor 2, resulting in decreased release of calcium from the sarcoplasmic reticulum [11]. These findings suggest that *TET2* mutations may impact both vascular inflammation and cardiac electrophysiology.

Intriguingly, *TET2* mutations may confer therapeutic sensitivity to anti-inflammatory interventions. This phenomenon is exemplified by canakinumab, an IL-1β inhibitor, which reduces MACE in post-myocardial infarction patients with elevated high-sensitivity C-reactive protein (hs-CRP) [112]. Notably, a prespecified genomic substudy revealed that *TET2*-mutant carriers exhibited a greater reduction in MACE risk (62%) with canakinumab therapy compared to non-*TET2* CHIP carriers (18% risk reduction) [113]. Supporting evidence from mouse models indicates that *TET2* deficiency promotes NLRP3 inflammasome activation, enhancing IL-1β secretion and atherosclerosis progression, which is ameliorated by canakinumab [41]. Notably, *TET2*-mutant carriers derive significantly greater benefit from canakinumab than normal controls, likely due to amplified inflammatory pathways in CHIP carriers [113, 114]. Future studies should explore gene-specific responses to other anti-inflammatory agents.

#### *DNMT3A and *CVD*s*

*DNMT3A* mutations have also been implicated in CVDs through aberrant inflammatory gene regulation. Monocytes and macrophages harboring *DNMT3A* mutations show upregulated expression of proinflammatory cytokines such as IL-1β, IL-6, IL-8, C–C motif chemokine ligand (CCL)3, and CCL4 [115]. The findings were also reconfirmed through the mouse models with transplanted *DNMT3A*^−/−^ HSCs, which suggested that *DNMT3A* depletion may serve as an inflammatory driving force for increased expression of proinflammatory cytokines [116]. Chronic inflammation drives cardiovascular pathogenesis through multiple mechanisms, including cytokine-mediated endothelial injury and oxidative stress-induced endothelial dysfunction, both of which accelerate atherosclerotic plaque formation [117, 118]. This process results in the buildup of unstable plaques. These deposits can also rupture, leading to blood clots that may cause heart attacks or strokes. Additionally, systemic inflammation from conditions like arthritis can impact the cardiovascular system through these very mechanisms [117, 118]. Hence, the altered functions of *DNMT3A* could potentially interfere with the inflammatory responses of immune cells, promoting the overproduction of these cytokines and becoming a causal link to CVDs. Moreover, DNA methylation of DNMT3A is also associated with an increased risk of hospitalization or death in patients with heart failure secondary to atherosclerotic CVD [119, 120].

Recent studies suggest that *TET2* and *DNMT3A* mutations converge on shared inflammatory pathways [37, 121, 122]. Deletion of either gene may promote a distinct population of adventitial macrophages with combined features of resident immune cells and inflammatory cytokine producers [123]. In an angiotensin II-induced mouse model of cardiac hypertrophy, double knockout of *TET2* and *DNMT3A* in HSPCs appeared to exacerbate cardiac hypertrophy, impair systolic function, and increase myocardial and renal fibrosis, suggesting that these CHIP mutations can potentiate heart failure and multiorgan dysfunction [122].

Although the precise mechanisms linking CHIP to CVDs remain incompletely defined, accumulating evidence suggests a central role of myeloid cell reprogramming in driving systemic and local inflammation [20, 41]. Therefore, individuals with CHIP mutations require regular cardiovascular monitoring and targeted clinical management.

## CHIP and metabolic diseases

### CHIP and T2DM

As previously noted, CHIP may be associated with an elevated risk of CVDs and increased mortality. Given that T2DM is a well-recognized risk factor for both CVDs and atherosclerosis, growing evidence suggests that T2DM may also be linked to CHIP, further supported by the indication of an association between T2DM and an increased risk of hematologic malignancies, such as lymphoma, leukemia, and myeloma [124]. Jaiswal et al. [6] reported that patients with diabetes are 1.3 times more likely to carry CHIP-related mutations. A large prospective cohort and genomic data from Tobias et al. [27] further support this association, revealing a 23% elevated T2DM risk in CHIP carriers over a decade-long follow-up, with those harboring *TET2* and *ASXL1* mutations conferring the highest susceptibility (Table 1). However, whether CHIP acts as a causal driver or merely a biomarker of underlying metabolic stress remains unclear.

Obesity, a key risk factor for T2DM [125], is also associated with an increased prevalence of CHIP [126, 127]. Studies from the UK Biobank and other cohorts have reported higher CHIP frequency and VAF among obese individuals, suggesting that metabolic dysregulation may promote clonal expansion [126]. This raises an important question regarding whether CHIP is an independent risk factor for metabolic disease or reflects pre-existing inflammation and metabolic aging. Beyond individual-level associations, obesity prevalence differs substantially across populations, raising the possibility that interethnic variation in metabolic traits (e.g., adiposity, insulin resistance, lipid profile) could contribute to differences in CHIP prevalence [128]. Nonetheless, direct evidence for such population-level effects is lacking, and future studies integrating harmonized genomic and metabolic profiling across diverse cohorts are warranted to clarify these relationships.

T2DM is increasingly recognized as a chronic inflammatory disease, where proinflammatory cytokines from macrophages and other tissues contribute to its development [129]. CHIP, driven by somatic mutations in HSCs, may link age-related alterations in the innate immune system to chronic low-grade inflammation [130, 131]. In this context, metabolic conditions such as obesity provide a proinflammatory milieu that can further interact with CHIP. The inflammatory landscape associated with obesity, marked by elevated IL-1β, IL-6, and TNF-α, may enhance the fitness of CHIP-mutant clones. Once expanded, these mutant clones may in turn amplify systemic inflammation, thereby reinforcing the inflammatory state. This establishes a feedforward loop that exacerbates metabolic dysfunction [126]. Supporting this, mouse models with *TET2*-deficient CH (a CHIP model) show exacerbated insulin resistance in aged and obese mice, potentially mediated through upregulation of the proinflammatory cytokine IL-1β in white adipose tissue [76]. Since insulin resistance is a significant pathogenic factor in T2DM, it can be inferred that CHIP, at least when driven by *TET2* mutations, is linked to the development of T2DM. This aligns with clinical observations by Kim et al. [132], which identified CHIP as a potential risk factor for T2DM and found that this association was particularly pronounced among individuals with elevated low-density lipoprotein cholesterol. Their study proposes that CHIP may synergize with elevated low-density lipoprotein cholesterol to promote T2DM progression by activating macrophages via the NLRP3 inflammasome and stimulating IL-1β release. Consistently, Bonnefond et al. [133] reported that T2DM patients with clonal mosaicism were more prone to vascular complications, providing clinical evidence that clonal expansions may aggravate diabetes-related vascular pathology. In this context, chronic inflammation driven by CH may further exacerbate vascular injury and accelerate atherosclerosis in diabetic individuals.

In contrast, conflicting evidence complicates this narrative. Recent evidence has failed to demonstrate a consistent link between CHIP and diabetes-related complications. Unlike other known risk factors such as albuminuria, heart failure, smoking, and elevated microinflammation, CHIP was not found to be connected to incident and progressive diabetic kidney disease [134]. Moreover, in a study of 294 patients, those without diabetic peripheral neuropathy (DPN) exhibited a higher prevalence of CHIP compared to those with DPN [135]. This discrepancy might be explained by the limited sample size for DPN (*n* = 113), highlighting the need for further research in larger cohorts to clarify this relationship. Given these mixed findings, we propose that CHIP is best conceptualized as a potential amplifier of pre-existing inflammatory and metabolic stress, rather than a sole initiator. Its contribution may depend on the specific driver mutation, degree of clonal expansion, and the presence of comorbidities such as obesity or dyslipidemia.

### CHIP and osteoporosis

Similar to CHIP, osteoporosis is an age-related disorder characterized by an imbalance between osteoblast-mediated bone formation and osteoclast-driven bone resorption [136]. CHIP-associated mutations, particularly in *DNMT3A* and *TET2*, drive sustained production of proinflammatory cytokines (e.g., IL-6, IL-1β) [20, 137, 138]. These cytokines and other immune-regulatory molecules not only contribute to cardiovascular and metabolic disorders but also disrupt bone homeostasis by modulating osteoclast and osteoblast activity [136, 139]. Epidemiological studies reveal that CHIP carriers face a 1.44-fold increased risk of osteoporosis compared to those without CHIP (Table 1) [77], with *DNMT3A* mutations showing the strongest association with reduced bone mineral density [83, 140]. Mechanistically, murine models demonstrate that *DNMT3A*-mutant macrophages secrete excess IL-20, directly enhancing osteoclast activity and accelerating bone loss [77].

Whether CHIP is a contributor to osteoporosis or merely reflects systemic inflammaging remains uncertain; however, the convergence of epidemiological and mechanistic evidence suggests a plausible pathogenic role. Preclinical and clinical validation of CHIP-targeted anti-inflammatory interventions in osteoporosis remains an important avenue for future research.

### CHIP and gount

Gout is an inflammatory arthritis associated with hyperuricemia, which is driven by the deposition of monosodium urate (MSU) crystals that activate the NLRP3 inflammasome and promote IL-1β secretion [141]. Emerging evidence suggests a mechanistic link between CHIP and gout through shared inflammatory pathways [142]. A biobank-based study identified *TET2* mutation-driven CHIP as a potential risk factor for gout, with carriers exhibiting heightened susceptibility to MSU crystal-induced inflammation (Table 1) [26]. Mechanistic support comes from murine models. *TET2* knockout mouse models exhibited heightened inflammatory responses upon MSU crystal stimulation, characterized by increased macrophage-derived IL-1β secretion, underscoring the contribution of CHIP to gout pathogenesis. Furthermore, a recent 2-sample Mendelian randomization study [142] suggested a potential causal relationship between CHIP and gout, highlighting that *DNMT3A* mutations may influence gout onset via chromatin remodeling and epigenetic modification mechanisms.

Collectively, these findings suggested that CHIP was an independent risk factor for chronic inflammation in gout and modulated innate immune responses. Targeting CHIP-associated inflammasome hyperactivation, such as through NLRP3 or IL-1β inhibitors, or applying epigenetic therapies tailored to specific mutations could provide new precision strategies for gout management. Nevertheless, such interventions remain largely theoretical at this stage, with no CHIP-targeted clinical strategies currently validated in gout.

## CHIP and kidney diseases

CHIP may influence kidney health across diverse populations. In the general population, CHIP was linked to an increased risk of a 30% estimated glomerular filtration rate (eGFR) decline and a greater risk of incident acute kidney injury (AKI) (Table 1) [143], with the latter risk being particularly pronounced in individuals with CHIP driven by mutations in genes other than *DNMT3A*, such as *TET2* and *JAK2* [29]. In *JAK2* V617F-mutant CHIP mice, tubular necrosis and fibrosis were more severe than in wild-type controls [144]. Clinically, individuals with large CHIP clones exhibited a 2.9-fold higher 5-year risk of kidney failure or ≥ 50% eGFR decline [29]. Among patients with advanced CKD, 23% harbored CHIP had a lower baseline eGFR than non-carriers [22.3 ml/(min·1.73 m^2^) vs. 28.2 ml/(min·1.73 m^2^)], after adjusting for age and sex, CHIP carriers faced a higher risk of progressing to end-stage renal disease or experiencing a ≥ 50% eGFR decline (Table 1). Despite more frequent use of erythropoiesis-stimulating agents, CHIP carriers remained more anemic (hemoglobin: 11.6 g/dl vs. 12.8 g/dl) and showed elevated serum ferritin and parathyroid hormone levels [75]. CHIP is also associated with monoclonal gammopathy of renal significance, a spectrum of kidney disorders caused by lymphoid-derived monoclonal immunoglobulins. Approximately 21% of light-chain amyloidosis patients harbor CHIP mutations [24]. In such cases, therapies targeting clonal plasma cells or B cells show therapeutic promise [145].

Beyond accelerating renal functional decline, emerging evidence suggests CHIP may modulate a range of CKD-related systemic complications. Patients with CKD, especially in its advanced stages, demonstrate increased susceptibility to cognitive decline and dementia [146]. However, CHIP carriers experienced 56% fewer attention deficits and 40% fewer executive‐function impairments. These protective effects were driven by small clones (VAF 2–8%), whereas larger clones showed no association [147]. This conclusion aligns with findings in the general population that CHIP reduces the risk of AD [148], providing new insights into the association between CHIP and neurocognitive outcomes in CKD patients and promising avenues to delay CKD-related cognitive decline.

CKD patients demonstrate higher incidence and mortality rates of cardiovascular events (heart failure, myocardial infarction, stroke) compared to the general population [149]. While the overall presence of CHIP in CKD patients without prior CVDs did not show a significant correlation with subclinical cardiac abnormalities, this broad assessment masks important genotype-specific risks. Notably, the impact of CHIP appears to be highly dependent on the specific driver mutation involved. For instance, *DNMT3A*-mutant clones were linked to a more than sixfold rise in MACE, which was attributed to hypomethylation-driven overexpression of the proinflammatory nuclear enriched abundant transcript 1 (*NEAT1*) gene [150]. A systematic meta-analysis demonstrates that CHIP clone size positively correlates with pan-arterial atherosclerotic events, with *TP53*-mutant clones showing preferential association with renal artery stenosis compared to other genotypic subgroups [73]. Renal artery stenosis reduces kidney perfusion, which may accelerate eGFR decline and progression to end-stage renal disease [151].

Across analyses of the US National Biobank and UK Biobank, kidney transplant recipients exhibited twofold higher odds of *TET2*-CHIP carriage compared with non-transplanted individuals [152]. Individuals who underwent transplantation before biobank enrollment showed a similarly elevated *TET2*-CHIP rate (*OR* = 1.90). Interestingly, this pattern is not observed in individuals who underwent transplantation after enrollment, indicating that the timing of transplantation relative to CHIP assessment may impact the detection of such mutations. The underlying mechanisms driving this selective enrichment remain unclear and warrant further investigation.

The clonal expansion characteristic of CHIP may generate mutant macrophages with excessive production of proinflammatory cytokines (IL-1β, IL-6, TNF-α). These mediators may induce tubular epithelial cell injury, myofibroblast activation, and epigenetic silencing of *Klotho*, a key protective factor. Murine models demonstrate that such an inflammatory milieu induces DNA hypermethylation and histone modification at the *Klotho* locus, thereby accelerating renal fibrogenesis [24]. Moreover, murine studies support a causal role for CHIP in kidney injury, as evidenced by bone marrow transplantation studies using *TET2*-deficient donors into *LDLR⁻/⁻* recipients, which showed glomerulosclerosis with foam cell accumulation [153]. Furthermore, mice carrying *DNMT3A* loss-of-function mutations developed exacerbated glomerulosclerosis and interstitial fibrosis compared to wild-type counterparts after angiotensin II administration [20, 122]. In humans, the link between CHIP and renal decline is less clear. Although Vlasschaert et al. [75] reported elevated risks of renal function deterioration in CHIP carriers, an independent investigation in diabetic nephropathy cohorts found no significant association [154]. Further research is needed to reconcile these conflicting results.

There is no approved therapy specifically for CHIP or CHIP‐associated CKD. However, preclinical studies suggest several promising approaches: high‐dose vitamin C to restore TET2 activity [24], rapamycin‐mediated mTOR inhibition to curb HSC expansion in *ASXL1*‐mutant models [47], blockade of the IL-1β/NLRP3 inflammasome to reduce CHIP-driven inflammation [111] combined with renin-angiotensin system and sodium-glucose cotransporter 2 (SGLT2) inhibitors to slow CKD progression [155, 156] and epigenetic upregulation of *Klotho* by suppressing miR-199a-5p released from injured tubular cells [157]. These mechanistic insights may provide a foundational framework for developing future diagnostic and therapeutic strategies for CHIP-associated CKD.

## CHIP and chronic infection

The study by Hormaechea-Agulla et al. [158] demonstrates that chronic infection can drive the clonal expansion of *DNMT3A* loss-of-function HSCs via the interferon-gamma (IFN-γ) signaling pathway. Activation of this pathway may enhance immune activity and exacerbate chronic inflammation, potentially giving mutated HSCs a competitive advantage within the bone marrow niche and contributing to CHIP development. Notably, somatic mutations in HSPCs may exert systemic effects by promoting the release of inflammatory mediators. In particular, *DNMT3A* appears to play a key role in regulating immune responses to infection and inflammation. These findings indicate that CHIP may not only result from chronic infection but could also actively contribute to disease progression in the context of persistent infection or prolonged immune activation [158]. For instance, CHIP has been associated with increased morbidity in individuals infected with various pathogens, including human immunodeficiency virus (HIV) and coronavirus disease 2019 (COVID-19) [31, 107, 159, 160]. In CHIP carriers, exaggerated or dysregulated inflammatory responses could worsen the clinical course of infections and potentially increase the risk of complications such as cardiovascular events or secondary infections.

### CHIP and HIV

One notable example of CHIP’s interaction with infectious diseases is HIV/acquired immune deficiency syndrome ​​(AIDS). In people living with HIV (PLWH), persistent immune activation and chronic inflammation may accelerate the development and clonal expansion of CHIP. Multiple studies, including the Swiss HIV cohort (Table 1), Age-Related Clonal Hematopoiesis in an HIV Evaluation cohort (ARCHIVE), and Copenhagen studies, reported a twofold increased CHIP prevalence in PLWH compared to matched controls, with the most frequently detected mutations including *DNMT3A*, *TET2*, and *ASXL1* [31, 161, 162]. Further insights from the REPRIEVE trial’s global cohort data suggest that several HIV-related factors, including advancing age, low CD4^+^ T cell counts, and smoking history, as well as lifestyle and environmental exposures, may contribute to CHIP emergence, compounding long-term health risks, especially cardiovascular complications, in PLWH [31, 163]. Similarly, Rocco et al. [79] proposed a bidirectional relationship between immunodeficiency and CHIP: advanced HIV (characterized by lower CD4^+^ T cell counts) correlates with higher CHIP prevalence, and the presence of opportunistic infections or inflammatory syndromes may further reinforce clonal expansion [79]. This suggests that unresolved pathogen burden and cytokine dysregulation could create a self-sustaining inflammatory loop that favors CHIP persistence. Screening PLWH for CHIP, especially those with persistent immune activation or poor treatment response, may enable personalized interventions targeting inflammatory pathways.

### CHIP and COVID-19

Since 2020, COVID-19 has emerged as the most widespread infectious disease globally. Population-based studies demonstrated that CHIP-positive individuals are at nearly twofold higher risk of hospitalization, mechanical ventilation, or death from COVID-19 (Table 1), even after adjustment for comorbidities such as hypertension, obesity, and diabetes [81, 159, 160]. Mechanistic studies have identified hyperactive inflammation as a key contributor. For example, Duployez et al. [164] assessed CHIP-related clinical and biological features in patients with severe COVID-19 and observed that *TET2* and *DNMT3A* mutations were associated with amplified inflammatory responses, which may exacerbate disease progression. Increased CHIP prevalence in hospitalized COVID-19 patients compared to age-matched controls further supports its role as a marker of host vulnerability [80]. Additionally, CHIP has been linked to increased susceptibility to other infections, such as *Clostridioides difficile* and *Streptococcus*/*Enterococcus*, likely due to the same underlying mechanisms of persistent low-grade inflammation and dysregulated immune responses that also contribute to worse COVID-19 outcomes. Overall, CHIP may influence COVID-19 severity through inflammatory dysregulation, but further studies are needed to clarify its clinical relevance and potential utility in patient management.

### Shared mechanisms and inflammatory signaling

Many of CHIP’s pathological effects across infections and chronic diseases converge on common inflammatory pathways. Mutations in *TET2*, *DNMT3A*, and *ASXL1* skew hematopoietic differentiation and enhance myeloid-driven cytokine production, particularly IL-1β and IL-6 [41, 77, 137]. These cytokines are central to the pathogenesis of infections (e.g., COVID-19 cytokine storm), as well as metabolic diseases (e.g., insulin resistance), liver fibrosis, and airway inflammation (e.g., COPD) [76, 132, 164–166]. CHIP-derived macrophages and neutrophils show heightened inflammasome activation and impaired resolution of inflammation, leading to tissue injury and impaired host defense [132, 166]. In infectious diseases, this may result in prolonged pathogen burden; in metabolic disease and COPD, it promotes sterile inflammation and organ damage [26, 77, 163]. Thus, CHIP acts not merely as a passive biomarker but as an amplifier of inflammatory injury.

Given the recurring involvement of the NLRP3/IL-1β axis, future therapies targeting shared signaling hubs could offer broad benefits across multiple CHIP-associated conditions. Notably, beyond its role in infections, CHIP also impacts chronic liver disease, COPD, neurodegeneration, and periodontitis, largely through shared inflammatory pathways. The following sections describe the evidence supporting CHIP’s involvement in each of these conditions.

## CHIP and other diseases

### CHIP and liver disease

Chronic liver diseases are characterized by persistent inflammation and progressive fibrosis over time. Emerging evidence suggests that CHIP may contribute to the progression of chronic liver disease and hepatic oncogenesis [30, 165, 167]. Notably, while CHIP with VAF < 10% appears to have no significant association with liver pathology, individuals with CHIP at VAF ≥ 10% may have a twofold increased risk of chronic liver disease, as shown across 4 independent cohorts (Table 1) [30]. Preclinical bone marrow transplantation models corroborate that *TET2*-mutant CHIP promotes hepatic inflammation and fibrosis through activating the NLRP3 inflammasome and downstream IL-1β and IL-6 signaling pathways [165].

In addition to chronic liver injury, CHIP’s oncogenic role is further highlighted in metabolic dysfunction-associated steatotic liver disease (MASLD). A European cohort study identified CHIP in 13% of MASLD patients with frequent mutations in *DNMT3A*, *TET2*, *TP53*, and *ASXL1*. After adjusting for covariates such as sex, diabetes status, polygenic risk scores, and cirrhosis, CHIP was associated with a twofold increased risk of hepatocellular carcinoma (HCC) [167]. Specifically, *TET2*-mutant CHIP conferred the highest risk, while *DNMT3A*-mutant CHIP did not show a significant association with chronic liver disease progression or HCC risk. These studies collectively highlight CHIP as a potential modifier of liver disease susceptibility and progression.

### CHIP and COPD

COPD is a progressive inflammatory disorder characterized by persistent respiratory symptoms, irreversible airflow limitation, and emphysema [168]. This condition is increasingly linked to CHIP. In a study conducted by Miller et al. [14], the risks of COPD were much higher in individuals with CHIP than those without CHIP (1.6 times in moderate-to-severe COPD and 2.2 times in severe or very severe COPD) (Table 1). In murine models, the inactivation of *TET2* in hematopoietic cells amplifies interferon-mediated inflammation, accelerating emphysema progression [14]. Similarly, *DNMT3A*-mutant macrophages exhibit IL-6 hypersecretion, driving airway inflammation and pulmonary function decline [78].

Recent data suggest CHIP may also interact with smoking to influence disease risk and progression [12, 166]. *ASXL1* mutations were more prevalent among current smokers with COPD compared to non-smokers [12, 166]. Strikingly, *ASXL1*-mutated CHIP was more strongly associated with current smoking than prior smoking history, suggesting that ongoing tobacco exposure may promote clonal selection [166]. Moreover, CHIP mutations were linked to increased frequency of acute COPD exacerbations, indicating that CHIP may influence both disease susceptibility and clinical severity [166]. Thus, advancing research into CHIP’s mechanisms in lung diseases, especially its interaction with COPD, may hold significant potential for improving patient management and treatment approaches.

### CHIP and neurodegenerative diseases

Notably, while CHIP-associated chronic inflammation typically contributes to disease progression in multiple organ systems, its role in neurodegenerative diseases appears more complex. A UK Biobank cohort study revealed CHIP carriers faced elevated risks of neurodegenerative diseases overall, with *DNMT3A* mutations specifically associated with vascular neurodegeneration and *SRSF2* mutations linked to increased susceptibility to amyotrophic lateral sclerosis (Table 1) [82]. These associations are consistent with the proinflammatory and vasculopathic phenotype associated with CHIP.

Interestingly, CHIP has been unexpectedly associated with a reduced risk of AD, a finding that challenges the prevailing narrative of CHIP as uniformly detrimental [148]. A meta-analysis of the Alzheimer’s Disease Sequencing Project (ADSP), Cardiovascular Health Study (CHS), and Framingham Heart Study (FHS) cohorts showed that CHIP carriers had a significantly lower risk of AD dementia. Moreover, brain autopsy data demonstrated that individuals with CHIP exhibited fewer β-amyloid plaques and neurofibrillary tangles on postmortem examination [148]. Mendelian randomization analyses further supported a potential causal relationship between CHIP and reduced AD risk. Mechanistically, this paradoxical effect may reflect a unique aspect of CHIP-driven immune remodeling in the central nervous system. One compelling hypothesis posits that peripheral myeloid cells derived from CHIP clones may infiltrate the brain, displace dysfunctional aging microglia, and restore phagocytic clearance of β-amyloid aggregates [148, 169]. This concept challenges the otherwise deleterious view of CHIP, suggesting that in certain contexts, CH may confer neuroprotective benefits by restoring immune surveillance within the central nervous system.

However, a South Korean positron emission tomography-imaging study found no association between CHIP and cerebral β-amyloid deposition [170]. Such divergent findings may stem from methodological factors, including small sample size, population homogeneity, and limited imaging sensitivity. This discrepancy underscores the need for longitudinal, multimodal studies integrating CHIP mutational profiles, neuroimaging biomarkers, and histopathological validation.

In summary, while CHIP appears to confer increased susceptibility to certain neurodegenerative diseases, it may paradoxically offer neuroprotective effects in AD. If confirmed, this inverse association could open new avenues for AD prevention or treatment by harnessing CHIP-like immune remodeling.

### CHIP and periodontitis

Periodontitis is a prevalent chronic inflammatory disease involving local and systemic immune responses, with susceptibility increasing as individuals age [171, 172]. As a systemic driver of chronic low-grade inflammation, CHIP may amplify both systemic and local immune responses, thereby predisposing individuals to periodontal tissue destruction [130, 131]. In a cohort study of 4946 adults, *DNMT3A*-mutated CHIP was linked to more severe gingival inflammation and alveolar bone loss (Table 1) [83]. Mechanistic investigations in *DNMT3A*^R878H/+^ mouse models demonstrated that CHIP-derived myeloid cells exhibit hyperactive mTOR signaling, enhanced osteoclastogenesis, and elevated secretion of proinflammatory cytokines such as IL-23 and IL-17 [83]. Importantly, treatment with the mTOR inhibitor rapamycin mitigated these pathological features by suppressing the expansion of *DNMT3A*-mutant clones and reducing the infiltration of inflammatory leukocytes into periodontal tissues, suggesting CHIP-targeted immunomodulation may be a viable strategy in age-related disease [83].

## Future perspectives

CHIP is characterized by the presence of a genetically distinct subpopulation of blood cells derived from HSCs that carry leukemogenic somatic mutations. It has emerged as a critical link between aging, chronic inflammation, and the pathogenesis of diverse systemic diseases [130]. Initially considered a precancerous state preceding hematologic malignancies, CHIP is now increasingly implicated in a wide range of non-hematologic conditions. This review synthesizes current evidence across 12 major disease categories, highlighting both shared mechanisms and unresolved complexities underlying CHIP-associated pathologies.

Shared inflammatory signaling pathways represent a unifying mechanism across these conditions. CHIP-associated mutations, such as in *TET2*, *DNMT3A*, and *ASXL1,* reshape the function of myeloid cells, leading to increased secretion of cytokines like IL-1β and IL-6, activation of the NLRP3 inflammasome, and systemic low-grade inflammation. Notably, some studies have found that diseases may assist CHIP clones in establishing dominance [126, 173]. For example, atherosclerosis promotes HSC proliferation, which accelerates mutant clone expansion. In the context of a systematic inflammation mediated by CVD, mutant clones with competitive advantages may expand faster, contributing to the development of CHIP [173]. Yet, it is a “chicken and egg” paradox about which comes first. CHIP mutant cells may accelerate disease development by increasing inflammatory factor levels [76]. In turn, diseases lead to the expansion of CHIP clones by altering the bone marrow microenvironment, which creates positive feedback and ultimately results in mutual progression [130, 173]. This positive feedback mechanism underscores a bidirectional relationship that remains inadequately studied, highlighting the need for further research into organ-specific interactions and underlying molecular mechanisms.

Remarkably, multisystem diseases often involve complex interactions, particularly in common metabolic disorders such as diabetes, which are frequently accompanied by multiple comorbidities [174]. This overlapping disease state complicates efforts to delineate CHIP’s specific role in individual conditions. For instance, CVD and diabetes are well-established risk factors for CKD [175], implying that the progression of these pre-existing conditions may partially mediate the potential association between CHIP and CKD. A critical knowledge gap remains in understanding the precise molecular mechanisms through which CHIP-driven chronic inflammation and immune dysregulation promote the concurrent development of cardiovascular, hepatic, and renal diseases, and whether CHIP acts as an independent driver of disease progression or primarily amplifies existing pathological processes. Moreover, the cumulative effects and interaction mechanisms of multiple CHIP-related mutations in individual patients remain insufficiently understood, particularly within the framework of gene-environment interactions. Environmental factors like infections, metabolic disorders, medications, and disease onset timing may influence CHIP-disease interactions through diverse biological pathways. Large-scale, long-term prospective cohort studies are urgently needed to better quantify the disease risks and clinical outcomes, and to disentangle its independent and synergistic effects in complex clinical settings.

In parallel, it is worth mentioning that Schenz et al. [80] reported that COVID-19 patients with CHIP exhibit age-dependent immune alterations: younger individuals tend to experience persistent lymphopenia, whereas older individuals show neutrophil expansion. These findings suggest that age significantly modulates the immunologic consequences of CHIP, potentially influencing both innate and adaptive immune responses. Age-related immunosenescence and dynamic immune remodeling may facilitate immune evasion by CHIP clones, while the accompanying chronic inflammatory milieu fosters a permissive environment for their clonal expansion [80, 158]. This complex interplay between aging, CHIP, and immune dysregulation warrants deeper investigation. Key questions include how CHIP-associated mutations shape the phenotype and function of myeloid cells (e.g., macrophages and neutrophils), influence lymphocyte activity, and modulate adaptive and antiviral immunity across different age groups. It also remains unclear whether CHIP impairs the resolution of viral infections or alters the balance between immune activation and exhaustion. Moreover, the possibility that anti-inflammatory therapies could inadvertently promote CHIP clonal dominance in immunocompromised individuals highlights the need for careful therapeutic evaluation in this context.

CHIP holds promise as a biomarker for risk stratification and therapeutic guidance, for example, in predicting cardiovascular risk among patients with chronic HIV infection [31, 163], or in identifying individuals with chronic liver disease at risk for progressive fibrosis [165] and patients with COPD at heightened risk of exacerbations [12, 166]. However, further specificity is required for clinical application. It remains unclear which CHIP mutations and VAF thresholds are most informative, and in which populations screening would be cost-effective. Anti-inflammatory treatments such as NLRP3 inhibition or IL-1β blockade aim to alleviate CHIP-associated inflammation, but they target downstream consequences rather than the initiating cause, mutant hematopoietic clones. More durable strategies may involve directly targeting clonal drivers. A CRISPR/Cas9-engineered *TET2*-mutant rhesus macaque model recapitulated key features of human CHIP and demonstrated that IL-6 axis blockade could suppress mutant clonal expansion [176]. Vitamin C supplementation has been shown to enhance residual TET2 activity in *TET2-*mutant leukemia models, reversing aberrant DNA methylation and restraining leukemic stem cell proliferation, thereby delaying disease progression [177]. Additionally, metformin has been shown to suppress *DNMT3A* R882-mutant HSPC expansion and AML progression by modulating mitochondrial metabolism and restoring methylation balance [178, 179]. Although still at the preclinical stage, these approaches provide a conceptual framework for targeting CHIP at its source.

## Conclusions

In summary, CHIP links somatic evolution in hematopoiesis to systemic aging and chronic disease. Its full clinical utility depends on clarifying causal mechanisms, distinguishing driver mutations, quantifying risk, and developing mutation-specific therapies.

## Data Availability

Not applicable.

## Abbreviations

AD: Alzheimer’s disease
AF: Atrial fibrillation
AKI: Acute kidney injury
Akt/mTOR: Protein kinase B/mammalian target of rapamycin
AMI: Acute myocardial infarction
AML: Acute myeloid leukemia
ARIC: Atherosclerosis risk in communities
ASCVD: Atherosclerotic cardiovascular disease
ASXL1: Additional sex combs like 1
ATM: Ataxia telangiectasia mutated
BAP1: BRCA1-associated protein 1
BRCA: Breast cancer associated gene
CCL: C–C motif chemokine ligand
cfDNA: Cell-free DNA
CH: Clonal hematopoiesis
CHIP: Clonal hematopoiesis of indeterminate potential
CKD: Chronic kidney disease
CMD: Coronary microvascular dysfunction
COPD: Chronic obstructive pulmonary disease
COVID-19: Coronavirus disease 2019
CS: Cardiogenic shock
ctDNA: Circulating tumor DNA
CVDs: Cardiovascular diseases
CH-PD: CHIP with putative driver mutations
DNMT3A: DNA methyltransferase 3 alpha
DPN: Diabetic peripheral neuropathy
eGFR: Estimated glomerular filtration rate
GOLD: Global initiative on obstructive lung disease
HCC: Hepatocellular carcinoma
HFpEF: Heart failure with preserved ejection fraction
HIV: Human immunodeficiency virus
HR: Hazard ratio
HSCs: Hematopoietic stem cells
HSPCs: Hematopoietic stem/progenitor cells
IL: Interleukin
JAK2: Janus kinase 2
JAK-STAT: Janus kinase-signal transducer and activator of transcription
MSU: Monosodium urate
MACE: Major adverse cardiovascular events
MASLD: Metabolic dysfunction-associated steatotic liver disease
MGBB: Mass general brigham biobank
NGS: Next-generation sequencing
NLRP3: NLR family pyrin domain containing 3
NMSC: Non-melanoma skin cancer
OR: Odds ratio
PAD: Peripheral artery disease
PLWH: People living with HIV
SF3B1: Splicing factor 3B subunit 1
SRSF2: Serine and arginine rich splicing factor 2
U2AF1: U2 small nuclear RNA auxiliary factor 1
T2DM: Type 2 diabetes
TI-CH: Tumor-infiltrating clonal hematopoiesis
TET2: Tet methylcytosine dioxygenase 2
t-MN: Therapy-related myeloid neoplasms
TP53: Tumor protein p53
Tregs: Regulatory T cells
VAF: Variant allele frequency
